# Hand Book of the Practice of Medicine

**Published:** 1878-05

**Authors:** 


### Books Reviewed.

*Hand-Book of the Practice of Medicine.* By M. Charteris, M. D., Professor of Practice of Medicine, Anderson's College, Glasgow, &c. With Illustrations. Philadelphia: Lindsay & Blakiston, 1878. Buffalo: T. H. Butler. Price, $2,00: pp. 336.

This is the introduction to a series of small volumes by eminent authors for the use of students.

Although not sufficiently broad and suggestive to be relied upon for counsel in conducting difficult cases, it is a very good resumé of ordinary routine work. Its descriptions of disease are excellent, mode of practice recommended, Anglo-German; and the rationalé of things to be done, as a rule, not given. The antimonial treatment is named as being best in pneumonia, but the author says that "pneumonia occurring in the young or very old, is attended with great danger." We believe that American physicians use less antimony in this condition, and obtain better results.

Tr. perchloride of iron 30 minims every two hours in water, the author says, is the best internal remedy in diphtheria.

The illustrations are of the best, and the appendix of ninety-two formulæ will be useful for comparison in study, and suggestion in practice. M. B. M.

